# Syndrome de Cushing pendant la grossesse: à propos d'un cas d'adénome surrénalien

**DOI:** 10.11604/pamj.2015.21.81.6775

**Published:** 2015-06-02

**Authors:** Amal Touiti, Ghizlane El Mghari, Nawal El Ansari

**Affiliations:** 1 Service d'Endocrinologie, Diabétologie et Maladies Métaboliques, Hôpital Arrazi, CHU Mohammed VI, Laboratoire de Recherche de Pneumo-cardio-Immunopathologie et Métabolisme (PCIM), Faculté de Médecine et de Pharmacie de Marrakech, Université Cadi Ayad, Marrakech

**Keywords:** syndrome de Cushing, grossesse, adénome surrénalien, Cushing syndrome, pregnancy, adrenal adenoma

## Abstract

Nous rapportons le cas d'une patiente de 28 ans qui a présenté durant le deuxième trimestre de la grossesse une prise excessive du poids avec bouffissure du visage et apparition de vergetures pourpres au niveau de l'abdomen. Le diagnostic positif de syndrome de Cushing a été posé après l'accouchement devant deux CLU élevés et un test de freinage minute négatif. L’étiologie retenue était un adénome cortisolique. La prise en charge a consisté en une surrénalectomie gauche.

## Introduction

Le syndrome de cushing est une affection rare mais grave qui peut engager le pronostic vital indépendamment de sa cause. Son incidence est de 1 nouveau cas par an par million d´habitants [1]. Le syndrome de Cushing (SC) est plus rarement encore retrouvé pendant la grossesse car l'hypercorticisme est souvent associé à une insuffisance gonadotrope responsable le plus souvent d'une infertilité [1]. Le premier cas de syndrome de cushing pendant la grossesse a été décrit en 1953 par Hunt et Mc Conahey [2]. Depuis, peu de cas ont été rapportés dans la littérature à partir de série de cas [3]. Moins de 150 cas approximativement ont été decrits dans la littérature [4]. L´âge gestationnel moyen au diagnostic est de 18 semaines d´aménorrhée (SA) [1, 5]. Le diagnostic de syndrome de Cushing pendant la grossesse est délicat et difficile vu les modifications physiologiques de l'axe corticotrope survenues au cours de la grossesse. La survenue du syndrome de cushing au cours de la grossesse peut être associée à un risque accru de morbi mortalité fœto-maternelle [3]. Nous rapportons à travers cette observation le cas d'un syndrome de Cushing survenu au cours de la grossesse.

## Patient et observation

**Figure 1:**
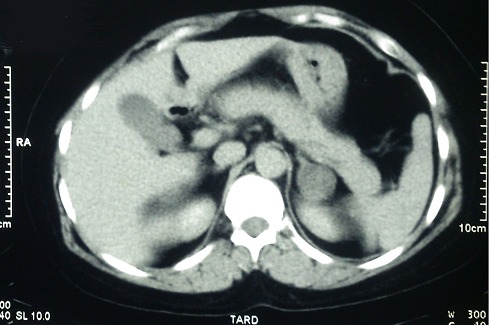
TDM surrénalienne, coupe transversale. Masse de la loge surrénalienne gauche, bien limitée mesurant 26x22mm

Il s'agit d'une patiente âgée de 29 ans sans antécédents pathologiques notamment pas de diabète connu ni hypertension artérielle (HTA) ni notion de prise de corticothérapie au long cours. Le début de la symptomatologie remonte à la dernière grossesse au 2 ^ème^ trimestre par l'installation d'une prise excessive du poids sans modifications des habitudes alimentaires avec bouffissure du visage, érythrose des pommettes et l'apparition de vergetures pourpres au niveau de l'abdomen avec fatigabilité musculaire et asthénie. La grossesse s'est déroulée sans incidents avec un accouchement médicalisé et un poids de naissance à 3500g. L’évolution après l'accouchement a été marquée par la persistance du surpoids avec une légère régression des vergetures pourpres. Le diagnostic de cushing a été posé cliniquement devant la présence de signes d'hypercorticisme à type d'obésité faciotronculaire, un surpoids avec IMC à 28kg/m^2^, un visage lunaire bouffi erythrosique avec comblement des espaces suscalviculaires, une bosse de bison, des vergetures pourpres au niveau de l'abdomen et la racine des cuisses, avec signes de fragilité cutanée. Le diagnostic de l'hypercorticisme a été confirmé biologiquement par des CLU élevés à 2 reprises: 188ug/24h recontrolée à 206ug/24h avec test de freinage minute négatif avec Cortisolémie à 5µg/dl après freinage. Dans le cadre du bilan étiologique, un dosage de l'ACTH a été réalisé initialement était à 19 mg/l. un test de freinage fort était négatif. L'IRM hypothalamo-hypophysaire a montré un discret bombement du diaphragme sellaire du coté droit sans lésion décelable de microadénome. La TDM surrénalienne réalisée a mis en évidence une masse surrénalienne gauche dont l'aspect et la densité et la cinétique en faveur d'un adénome surrénalien de 26*22mm (Figure 1) Dans le cadre du bilan de retentissement, un holter tensionnel a mis en évidence une HTA diastolique nocturne, à l'ODM une ostéopénie, avec une Glycémie à jeun à 1,06 g/l et une HbA1c à 5,2%. Une surrénalectomie gauche a été réalisée par voie coelioscopique avec à l'examen anatomopathologique un aspect d'un adénome surrénalien sans emboles vasculaires ni invasion capsulaire. L’évolution a été marquée après la chirurgie par une régression de l'hypercorticisme, une perte de 10 kg sur un an et une normalisation de la tension artérielle. Une grossesse est programée chez la patiente.

## Discussion

La survenue de grossesse chez une patiente porteuse d'un syndrome de cushing est une association rare. l'hyppercorticisme et l'hyperandrogenie sont souvent associées à une inssifisance gonadotrope source de troubles du cycle et d'infertilité [2, 4]. La grossesse est associée en général à des modifications de l´axe hypothalamo-hypophysaire corticotrope maternel. L´augmentation de la sécrétion placentaire d´estrogènes stimule la sécrétion de CBG (cortisol binding globulin) du foie, augmentant la production de cortisol et le taux de cortisol lié à la CBG. De ce fait, le taux de cortisol circulant et le cortisol libre urinaire augmentent pendant la grossesse [1, 6]. Le diagnostic du syndrome de cushing chez la femme enceinte est difficile et délicat. Sur le plan clinique, Peu de signes diffèrent entre la femme enceinte et non enceinte [1]. Le syndrome de cushing peut passer inaperçue pendant la grossesse. En effet, la similitude des symptômes cliniques avec certaines manifestations physiologiques de la grossesse tels que l´apparition de vergetures abdominales et d´une prise de poids peut expliquer le retard diagnostique [6]. D'autre part certaines complications du syndrome du cushing peuvent également être attribuées à des complications de la grossesse: diabète gestationnel, pré éclampsie [7]. Par ailleurs, Dans notre observation, le diagnostic n'a été évoqué qu'après l´accouchement. La prise du poids et les vergetures étaient mise sur le compte de la grossesse. Le diagnostic positif du syndrome de cushing repose sur trois examens: le dosage du CLU des 24 heures, le test de freinage faible à la dexaméthasone ainsi que le dosage du cortisol de minuit ou test salivaire du cortisol. Le rythme circadien du cortisol disparaît lors du SC mais pas lors d´une grossesse normale. L´élévation de la cortisolémie de minuit peut aider à confirmer le SC chez la femme enceinte. La mesure du CLU, reflet direct du cortisol libre circulant, est le gold standard pour détecter l´hypercortisolémie. Pendant la grossesse, le CLU augmente au 2ème et 3ème trimestres [8]. Pendant la grossesse, il convient de ne tenir compte du CLU aux 2èmes et 3ème trimestres que pour des valeurs supérieures à 3 fois la norme [1]. Le diagnostic étiologique repose sur plusieurs examens complémentaires: le dosage de l´ACTH, Les tests de freinage fort à la dexamethasone, les tests dynamiques non invasifs à la CRH (corticotropin releasing hormone), à la desmopressine, le cathétérisme veineux du sinus pétreux. L´imagerie de référence est l´imagerie par résonance magnétique (IRM) hypophysaire et abdominopelvienne sans injection de gadolinium, préférée à la tomodensitométrie pendant la grossesse [1, 8]. Les étiologies du syndrome de cushing chez la femme enceinte sont dominées par les adénomes surrénaliens, (40 à 50% des cas) [4], comme c’était le cas chez notre patiente. Ceci peut etre expliqué par ceratis auteurs par l'eventualité de secretion minimie d'androgene par les adénomes cortisoliques rendant la grossesse possible, par ailleurs, en cas de maladie de cushing, l'hyperandrogenie et l'hyppercortisime bloquent l'ovulation, et la survenue d'une grossesse est alors tres rare [7].

La maladie de Cushing est plus rare pendant la grossesse (33% pour 122 grossesses) [2, 8]. Par ailleurs, plusieurs cas de syndromes de Cushing induits par la grossesse régressent spontanément dans le post-partum ont été décrits [7]. Ces derniers étayent l'hypothèse soit d'un abaissement du seuil de sensibilité des surrénales maternelles à leurs stimuli habituels soit d'une stimulation excessive par une molécule sécrétée par le placenta telle que l'ACTH placentaire ou le Corticotropin-releasing factor (CRF) placentaire. Chez notre patiente, l’étiologie retenue du syndrome de cushing était un adénome surrénalien confirmé sur l’étude anatomopathologique. Le syndrome de cushing est associé à un risque accru de morbidité materno fœtale. Les complications maternelles les plus fréquemment retrouvées sont l´hypertension artérielle, la prééclampsie, le diabète ou l´intolérance au glucose gestationnels. D'autres complications sont plus rarement associes: retards à la cicatrisation, des fractures, des troubles psychiatriques [1]. Par ailleurs, des complications fœtales sont dominées par la prématurité (43% des grossesses décrites), de fausses couches spontanées, de retards de croissance in utero et de décès néonataux [8]. Chez notre patiente, aucune complication n'a été détectée au cours de la grossesse, une grossesse est actuellement programmée. Le traitement a pour but de réduire la morbidité maternofoetale. La surrénalectomie pour les porteuses de tumeur surrénalienne peut être bénéfique avec des taux de naissance proches de 87% [1]. La chirurgie est le traitement de choix du SC pendant la grossesse, en dehors de la fin du 3e trimestre [9]. Le traitement médical reste un traitement de seconde intension. La métopirone est le traitement le plus utilisé sans effet secondaire sur la fonction hépatique maternelle ou le développement fœtal. Cependant, la métopirone peut exacerber une hypertension artérielle et favoriser la prééclampsie, ce qui doit limiter son utilisation. L'utilisation du kétoconazole doit être deconseillé du fait du passage transplacentaire et de son action tératogène et abortive. Le mitotane et l´aminoglutethimide sont contre-indiqués pendant la grossesse. En cas de maladie de Cushing (adénome corticotrope), le traitement de première intention sera l´exérèse de l´adénome par chirurgie hypophysaire par voie transsphénoïdale. S´il existe une extension suprasellaire trop importante, un abord par voie haute peut être nécessaire [7].

## Conclusion

Le syndrome de Cushing est rare durant la grossesse. Son diagnostic reste difficile et délicat chez la femme enceinte. Sur le plan clinique, peu de signes diffèrent entre la femme enceinte et non enceinte. Le diagnostic de syndrome de cushing est passé inaperçu chez notre malade L’étiologie la plus fréquente durant la grossesse est l'adénome surrénalien. La nécessité d'un diagnostic est justifiée par les complications materno-fœtales liée à cette pathologie.
